# The Dual Action of Epigallocatechin Gallate (EGCG), the Main Constituent of Green Tea, against the Deleterious Effects of Visible Light and Singlet Oxygen-Generating Conditions as Seen in Yeast Cells

**DOI:** 10.3390/molecules170910355

**Published:** 2012-08-29

**Authors:** Radu Mitrica, Ioana Dumitru, Lavinia L. Ruta, Augustin M. Ofiteru, Ileana C. Farcasanu

**Affiliations:** 1 Postgraduate Department of Biochemistry, University of Medicine & Pharmacy “Carol Davila”, Sos. Fundeni 258, Bucharest, Romania; Email: radu1100@yahoo.com; 2 Department of Organic Chemistry, Biochemistry and Catalysis, Faculty of Chemistry, University of Bucharest, Sos. Panduri 90-92, Bucharest, Romania; Email: ioanaa.dumitru@g.unibuc.ro (I.D.); lavinia.ruta@g.unibuc.ro (L.L.R.); augustin.ofiteru@g.unibuc.ro (A.M.O.)

**Keywords:** green tea, epigallocatechin gallate, *Saccharomyces cerevisiae*, visible light, singlet oxygen, UV-A

## Abstract

Green tea extracts (GTEs) as well as their main component, the polyphenol epigallocatechin gallate (EGCG), are known for their versatile antioxidant, antimicrobial, antitumoral or anti-inflammatory effects. In spite of the huge beneficial action, there is increasing evidence that under certain conditions green tea and its components can be detrimental to living organisms. Using *Saccharomyces cerevisiae* strains with various defects in the response to oxidative stress, we found that GTEs or EGCG act in synergy with visible light, exhibiting either deleterious or protective effects depending on the solvent employed. Similar synergistic effects could be observed under singlet oxygen-generating conditions, such as light exposure in the presence of photosensitizers or UV-A irradiation, therefore solvent variance may represent a powerful tool to modulate the preparation of green tea extracts, depending on the intended target.

## 1. Introduction

Antioxidants have been under the spotlight of research for many years, since it is widely believed that they have protective effects against the deleterious action of reactive oxygen species (ROSs) which evolve during aerobic metabolism. Green tea, one of the most widespread beverages in the World, is rich in polyphenols, out of which epigallocatechin gallate (EGCG) is by far the most important. Extensive studies on green tea polypehols have demonstrated preventive effects against heart diseases, diabetes, neurodegenerative disorders and cancer (for reviews, see [1,2,3,4]). Among the numerous mechanisms proposed for the chemoprotective effects of green tea and EGCG, the antioxidant and more recently the prooxidant activities are extensively considered as potential mechanisms of cancer prevention [5,6,7] or of anti-aging defense [8,9,10].

The budding yeast *Saccharomyces cerevisiae* has proved a very useful eukaryotic model for investigating the effects of small molecules at the cellular level; such studies not only verify the *in vitro* results of test-tube experiments of the protective effects, but also reveal the possible side effects and the products of their metabolism. Yeast is also an attractive alternative to mammalian cell lines, and especially to the controversial experiments on animals [11]. Yeast cells are thought to generate ROS through the same mechanisms as mammalian cells and express many of the same antioxidant factors [12,13], therefore they can provide a suitable system for the rapid detection of various anti- and/or pro-oxidants. 

EGCG is widely accepted as antioxidant, being a potent scavenger of various ROSs (for a review, see [14]). In contrast to the antioxidant activity, recent evidence revealed that EGCG has also pro-oxidant potential, by generating ROS *in vivo* [15]. In the few studies using yeast to characterize the EGCG activity, it was shown that strains of *S. cerevisiae* which lack either of the two main transcriptional factors involved in oxidative stress response (Yap1p and Skn7p) show increased sensitivity to GTEs [16]. Apparently, both GTEs and EGCG can act as pro-oxidants by generating endogenous H_2_O_2_ within the yeast cells, thus activating oxidative stress-related transcription factors in *S.* *cerevisiae* or *S. pombe* [15]. As Yap1p and Skn7p are the main transcription factors which regulate the response to oxidative stress in *S.* *cerevisiae* cells [17], in this study we used yeast mutants defective in *YAP1* or *SKN7* genes to further characterize the (non)protective action of GTEs and EGCG against the deleterious effects of visible light and singlet oxygen-generating agents. Using GTEs or EGCG in various polar solvents, both protective and adverse effects could be observed, depending on the nature of the solvent used. 

## 2. Results and Discussion

### 2.1. The Green Tea Extracts Exhibit Solvent-Dependent Effects on Yeast Cells Grown under Visible Light

**Figure 1:**
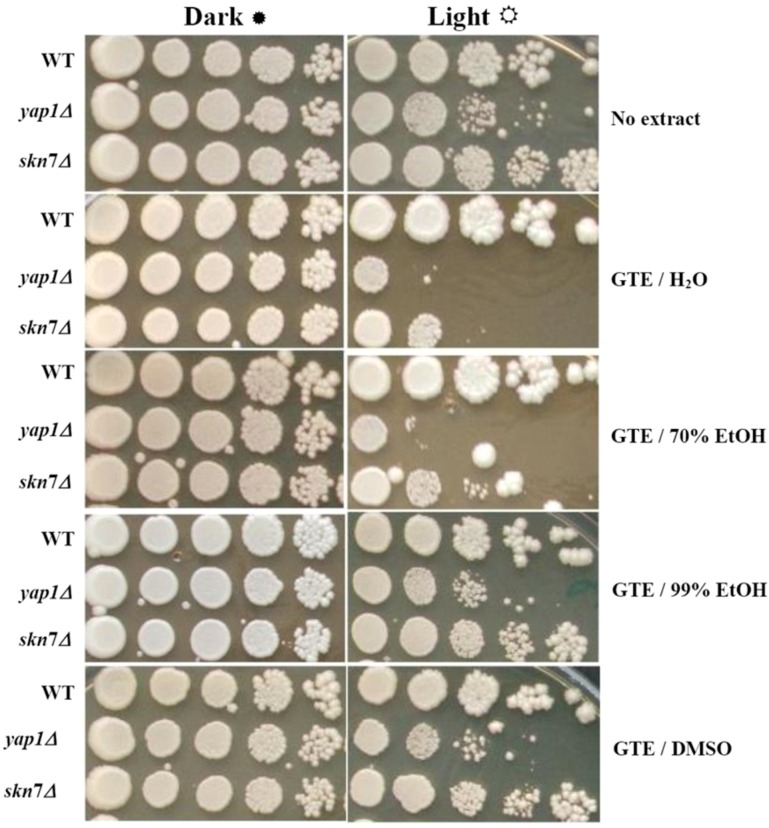
Effect of GTEs on the photosensitivity of yeast cells. Mid-log phase wild type (WT), yap1Δ and skn7Δ cells were spotted (approx 4 μL/spot) in ten-fold serial dilutions (from 10^7^ cells/mL, left, to 10^3^ cells/mL, right) onto YPD/agar containing 10 μL/mL extracts. Plates were photographed after three days incubation at 28 °C. The experiments were repeated on three different days and the results were similar.

**Figure 2:**
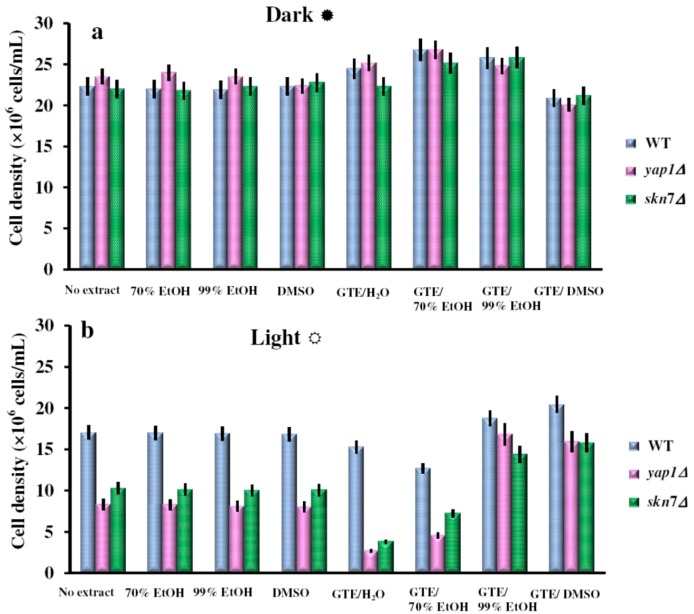
Effect of GTEs on photosensitivity of yeast cells. Overnight pre-cultures were inoculated in fresh YPD media at 2 × 10^5^ cells/mL, then cells were incubated with shaking (200 rpm) at 28 °C for two hours before GTEs were added (10 μL/mL). The cell growth in liquid media was detected after 24 h incubation in the dark (**a**) or under constant illumination with white light (**b**). For controls, the same amounts of carrier solvents as for the extract experiments were used. Each determination was repeated three times on different days, with no significant variations (*p* < 0.05). Values are expressed as the mean ± standard deviation (SD) of duplicate determinations of three independent experiments (n = 6).

The *S. cerevisiae* cells lacking the two main transcription factors involved in the oxidative stress response, Yap1p and Skn7p, are hypersensitive to ROS singlet oxygen (^1^O_2_) generated experimentally under exposure to visible light [18]. As GTEs were shown to have both pro-oxidant activity and deleterious effects on yeast cells which lack functional Yap1p [15,16], we decided to determine the influence of GTEs on the growth of yeast cells exposed to visible light. We obtained GTEs using five different polar extracting agents: water (GTE/H_2_O), 70% ethanol (GTE/70%EtOH), 99% ethanol (GTE/99%EtOH), and dimethylsulfoxide (GTE/DMSO). The extracts were obtained in the dark, under N_2_, using vacuum de-aerated solvents to avoid oxidation during extraction. The GTEs were assayed for total polyphenolic content (TPC) and were adjusted to the same TPC (2 mg gallic acid equivalents/mL) using the corresponding solvent. Serial dilutions of suspensions containing wild type cells, or cells with the genes *YAP1* or *SKN7* knocked out (*yap1Δ* or *skn7Δ* cells, respectively) were plated onto duplicate YPD agar plates containing 10 μL/mL of the GTEs obtained. The duplicate plates were incubated at 28 °C either in the dark or under constant illumination with white light (32 W/m^2^). Surprisingly, while having no obvious effect on cells grown in the dark, the extracts GTE/H_2_O and GTE/70%EtOH increased the sensitivity of *yap1Δ* or *skn7Δ* cells to illumination (Figure 1). As the ethanol concentration increased, the GTEs attenuated the deleterious effects of visible light on *yap1Δ* or *skn7Δ* cells, which were no longer recorded for GTE/99%EtOH (Figure 1). Similarly, sensitivity to visible light was not observed when cells were grown on media supplemented with GTE/DMSO (Figure 1). In the control experiment, it was noted that the extracting agents alone had no effect on cell growth (data not shown). The influence of GTEs on the growth of yeast cells was also monitored in liquid media (Figure 2). It was noted that both wild type and the oxidative stress-defective *yap1Δ* and *skn7Δ* cells grew better in the dark (Figure 2a). On the other hand, the exposure to visible light generally affected the cell growth, but *yap1Δ* and *skn7Δ* cells were more sensitive than the wild type (Figure 2b). In the control tests, it was noted that the carrier solvents alone did not significantly alter the growth of any mutant, either in the dark or under illumination (Figure 2a,b). As seen on agar plates (Figure 1), the GTEs prepared in H_2_O or ethanolic solutions (up to 70%, in water) augmented the photosensitivity of *yap1Δ* and *skn7Δ* cells, while GTEs obtained in 99% EtOH or DMSO exhibited a protective action upon these mutants (Figure 2b). Nevertheless, this difference was not the result of differentiated uptake modulated by the solvent, as the polyphenol uptakes by the cells were similar, irrespective of the extracting solvent used (data not shown). It is probable that the green tea polyphenols extracted by aqueous agents underwent oxidation more easily, thus exerting a pro-oxidative action upon the events imposed by visible light exposure. Another possible explanation is that the components of aqueous GTEs exerted a photosensitizing effect, thus augmenting the deleterious effect of visible light. In contrast, the extraction with the non-aqueous solvents (99% EtOH, DMSO) may have yielded compounds solvated by the less-polar-than-water molecules, which create a shielding effect on the polyphenols present in the GTEs, thus alleviating their pro-oxidant action.

### 2.2. Effect of the Green Tea Major Component EGCG on the Yeast Cells Grown under Illumination

Many of the biological properties of green tea are believed to be caused by the catechin fraction (an average 30% of the dry leaf weight), out of which EGCG is by far the most important compound, accounting for an estimative 50% of the catechin pool [19]. As such, we wondered if pure EGCG solved in the same agents used to prepare the GTEs would show similar synergism with the visible light against the yeast cells as the GTEs. 

To check this possibility, suspensions containing wild type, *yap1Δ* or *skn7Δ* cells were treated with EGCG dissolved in H_2_O, 70%EtOH, 99% EtOH or DMSO (corresponding to 10 μg/mL EGCG, final concentration; at this concentration EGCG was completely non-toxic to the yeast strains used, when grown in the dark). Cell suspensions were incubated with shaking in the dark or with constant illumination for 24 h, then cell growth was determined spectrophotometrically (OD_660_). 

**Figure 3:**
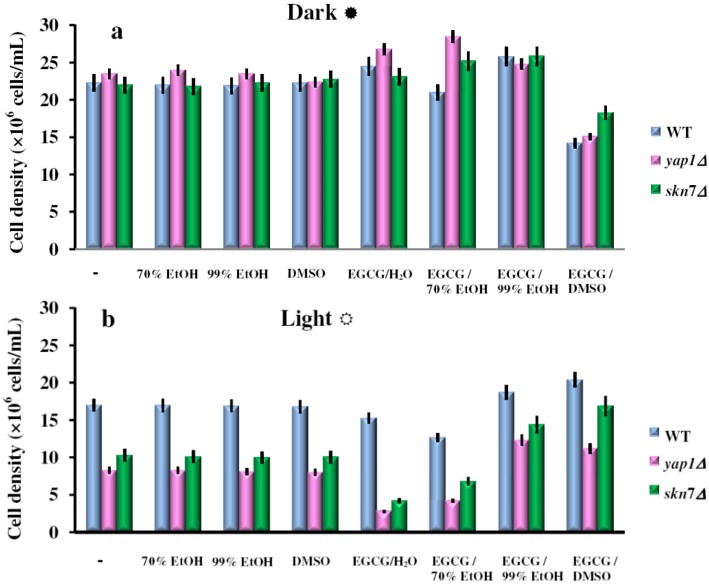
Effect of EGCG solutions on photosensitivity of yeast cells. Overnight pre-cultures were inoculated in fresh YPD media at 2 × 10^5^ cells/mL, then cells were incubated with shaking (200 rpm) at 28 °C for two hours before the EGCG solutions were added (final EGCG concentration, 10 μg/mL). The cell growth in liquid media was detected after 24 h incubation in the dark (**a**) or under constant illumination with white light (**b**). For controls, the same amount of the carrier solvents as in the EGCG experiments were used. Each determination was repeated three times on different days, with no significant variations (*p* < 0.05). Values are expressed as the mean ± standard deviation (SD) of duplicate determinations of three independent experiments (n = 6).

It was noted that similarly to GTEs, the EGCG aqueous or 70% EtOH solutions had deleterious effects on the cell growth, possible due to a pro-oxidant effect (Figure 3b). In contrast, the 99% EtOH or DMSO solutions had protective effects against the action of visible light (Figure 3b). In the control experiments, the carrier solvents did not significantly alter the cell growth (Figure 3a,b). These observations demonstrate that the effects of GTEs seen on cell growth under illumination (Figure 1 and Figure 2) are, at least in part, due to their main component, EGCG. It is largely believed that white light can exert various insults on cells during aerobic growth, mainly due to the generation of singlet oxygen (^1^O_2_), usually through energy transfer mediated by photosensitizers. Although generation of ^1^O_2_ was not measured directly, the presence of substances which are rich in mobile electrons, such as various dyes or polyphenols (including EGCG) are a token for ^1^O_2_ generation under visible light exposure [18,20]. Combination of green tea or EGCG with visible light irradiation was previously reported, with impressive impact on cell proliferation [21] and by varying the extraction solvent, this effect could be finely tuned. Also, recent studies indicate that chemo-optical synergism between 670-nm light and EGCG may represent a novel approach for addressing Alzheimer disease [22,23].

### 2.3. The Effect of the Green Tea Major Component EGCG on the Yeast Cells Grown under Illumination Depends on the Oxidative State of the Cell

**Figure 4:**
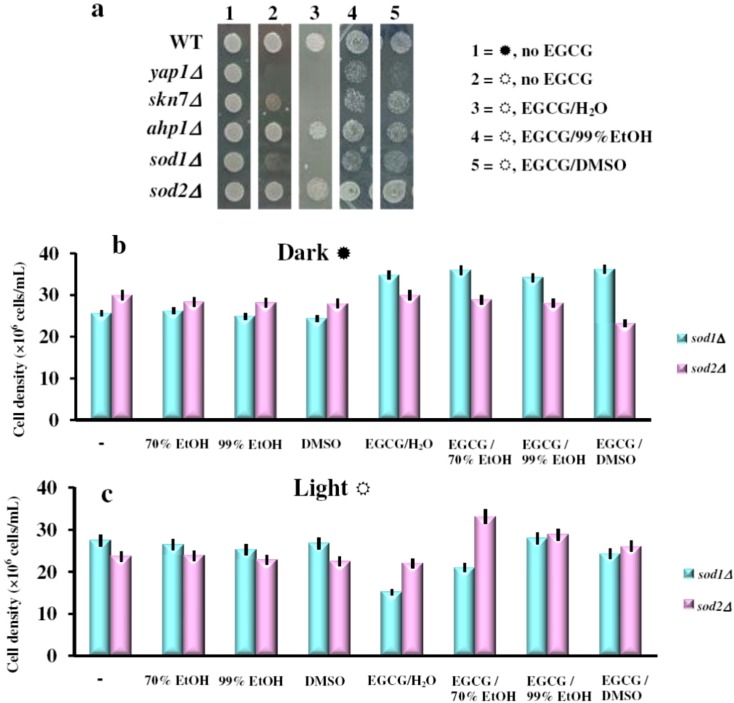
(**a**) Effect of EGCG on photosensitivity of yeast mutants with defects in response to oxidative stress. Cell suspensions (OD_660_ = 0.1) were stamped (4 μL/spot) onto YPD/agar plates containing or not EGCG (final concentration 10 μg/mL). Plates were photographed after three days incubation at 28 °C in the dark (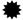) or under constant illumination (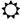). The experiments were repeated on three different days and the results were similar. (**b**) Effect of EGCG solutions on photosensitivity of *sod1Δ* and *sod2Δ* yeast knock-out mutants. Overnight pre-cultures were inoculated in fresh YPD media at 2 × 10^5^ cells/mL, then cells were incubated with shaking (200 rpm) at 28 °C for two hours before the EGCG solutions were added (final EGCG concentration, 10 μg/mL). The cell growth was detected after 24 h incubation in the dark (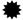) or under constant illumination with white light (**c**). For controls, the same amount of carrier solvents as in the EGCG experiments were used. Each determination was repeated three times on different days, with no significant variations (*p* < 0.05). Values are expressed as the mean ± standard deviation (SD) of duplicate determinations of three independent experiments (n = 6).

The ^1^O_2_ is an energetically excited state of oxygen and a powerful oxidant. Since ^1^O_2_ is believed to be generated during aerobic growth of yeast cells under illumination due to the presence of photosensitizers within the growth media [18,20], we tested the effect of EGCG solutions on the growth of other yeast mutants with defects in the oxidative stress defense (listed in Table 1). We found that besides *yap1Δ*, the cells most affected by EGCG under white light exposure were the *sod1Δ* cells. These cells lacked the *SOD1* gene encoding the Cu,Zn-superoxide dismutase, which predominantly localizes within the cytosol. In contrast, the *sod2Δ* cells which lack the mitochondrial Mn-superoxide dismutase were hardly affected by the EGCG under light (Figure 4a). Other mutants with defects in the oxidative stress response, such as *ahp1Δ*, were also unaffected by the presence of EGCG (Figure 4a). It is possible that in *sod1Δ* cells, the unscavenged superoxide augmented the cells’ sensitivity to EGCG in the presence of light. In *sod2Δ* cells, the superoxide is excessive only in the compartment where Sod2p is absent (*i.e.*, mitochondrion), while the visible light is probably prevented by the cytosol and by the mitochondrial membrane from reaching the mitochondrial matrix. The EGCG solutions described above were also tested on *sod1Δ* and *sod2Δ* cells in liquid cultures. Interestingly, while the effect of EGCG solutions followed the pattern described above (*i.e*., photosensitizers in the case of aqueous and 70% EtOH solutions, photoprotectants in the case of 99% EtOH or DMSO) all EGCG solutions seemed to be beneficial to *sod2Δ* cells grown in the dark (Figure 4b). 

**Table 1:** Effect of aqueous EGCG on the growth under white light exposure of various mutants with altered oxidative state. Strains were inoculated (initial cell density 5 × 10^5^ cells/mL) from overnight pre-cultures in YPD media containing 10 μg/mL EGCG (final concentration). Growth was assessed spectrophotometrically (OD_660_) after 16 h of incubation with shaking (200 rpm) at 28 °C, under constant illumination. The compound was considered to improve/impair the growth relatively to wild type when it caused increase/decrease of the cell density to more than 20%.

Strain used	Gene deleted	Effect of EGCG/H_2_O	Wild type function
Wild type	No	Control	
*sod1* *Δ*	*SOD1*	− ^a^	Cu,Zn-Superoxide dismutase
*sod2* *Δ*	*SOD2*	+ ^b^	Mn-Superoxide dismutase
*skn7* *Δ*	*SKN7*	−	Oxidative stress response
*yap1* *Δ*	*YAP1*	−	Oxidative stress response
*ahp1* *Δ*	*AHP1*	+	Thioredoxin peroxidase with alkyl hydroperoxide reductase activity controlling the levels of alkylhydoperoxides
*ccp1* *Δ*	*CCP1*	ND ^c^	Citocrome *c* peroxidase
*cta1* *Δ*	*CTA1*	ND	Catalase A (localized in peroxisomal and mitochondrial matrices)
*ctt1* *Δ*	*CTT1*	ND	Catalase T (localized in cytoplasm)
*gpx1* *Δ*	*GPX1*	ND	Glutathione peroxidase
*gpx2* *Δ*	*GPX2*	ND	Glutathione peroxidase
*hyr1* *Δ*	*HYR1*	−	Thiol peroxidase that functions as a hydroperoxide receptor to sense intracellular hydroperoxide levels and transduce a redox signal to the Yap1p transcription factor
*prx1* *Δ*	*PRX1*	ND	Mitochondrial peroxiredoxin with thioredoxin peroxidase activity
*tsa1* *Δ*	*TSA1*	−	Thioredoxin peroxidase which control the levels of H_2_O_2_. Gain-of-function mutant is resistant to alkyl hydroperoxides
*tsa2* *Δ*	*TSA2*	ND	Stress inducible cytoplasmic thioredoxin peroxidase

^a^ Impaired the growth; ^b^ Improved the growth; ^c^ No obvious effect on growth.

### 2.4. When in the Right Solvent, EGCG Can Rescue the Hypersensitive yap1Δ Cells from the Damaging Effect of ^1^O_2_

From previous research [18] as well as from the experiments described above it was clear that the *yap1Δ* cells were highly susceptible to combinations of ^1^O_2_ and EGCG. In aerobic cells, ^1^O_2_ evolves during enzymatic reduction or by lipid peroxydation [20,24]. 

**Figure 5:**
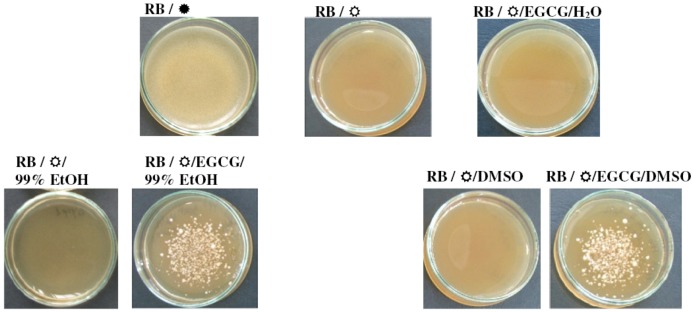
Effect of EGCG solutions on the ^1^O_2_-induced photosensitivity of *yap1Δ* cells. Mid-log phase cells were suspended at cell density of 1 × 10^5^ cells/mL in 1 mL of molten soft agar YPD (0.7% agar, 50 °C) containing 1.5 μM Rose Bengal (RB) and spread on YPD-agar plates (2 cm diameter) containing the same concentration of the chemical. After solidification of the top agar, EGCG solutions were added (0.1 mM, 15 μL) onto the surface of the top agar. The halo of the growth zone around the spotted sample was observed after three days incubation at 28 °C in the dark (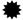) or under constant illumination with white light (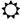). For controls, the same amount of the carrier solvents as in the EGCG experiments were used. The experiments were repeated on three different days and the results were similar. One representative set of plates is shown.

Additionally, ^1^O_2_ can be generated by photochemical reactions via energy transfer reactions from excited photosensitizers (e.g., exogenous dyes such as Rose Bengal, methylene blue or acridine orange) to molecular O_2_[20,24]. To see whether EGCG can rescue the *yap1Δ* photosensitivity towards Rose Bengal, aqueous, ethanolic or DMSO solutions of EGCG were placed onto cells spread on media containing 1.5 μM RB. We noted that the light-exposed *yap1Δ* cells died in the presence of RB, and that this sensitivity was rescued by placing EGCG solutions (100 μM, from 10 mM stock prepared in 99% EtOH or DMSO) on top of the cells. In contrast, the photosensitivity of *yap1Δ* cells to RB could not be alleviated by aqueous solutions of EGCG or by the carrier solvents (Figure 5).

### 2.5. The Effect of EGCG on the Yeast Cells Exposed to Ultraviolet A

In addition to visible light, ^1^O_2_ can be induced by exposure to ultraviolet A (UV-A). We therefore tested whether EGCG had any influence on cells exposed to UV-A irradiation. For this purpose, cell suspensions containing wild type, *yap1Δ* or *skn7Δ* cells were treated with EGCG dissolved in H_2_O, 70% EtOH, 99% EtOH or DMSO (corresponding to 10 μg/mL EGCG, final concentration).

**Figure 6:**
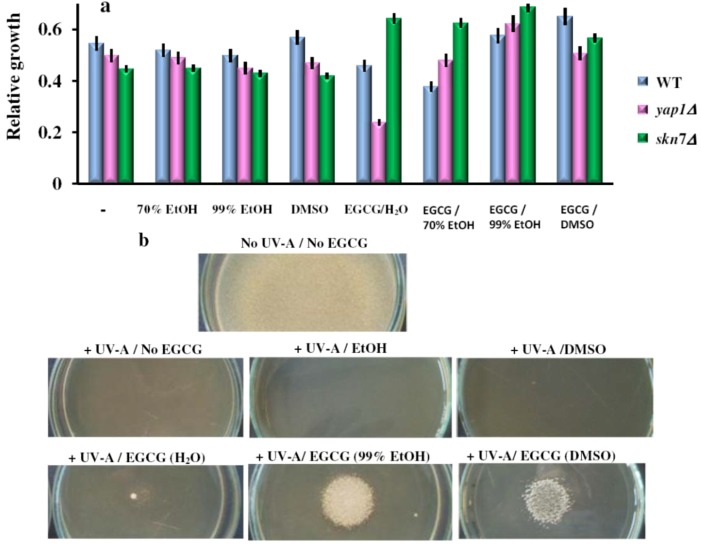
Effect of EGCG solutions on the UV-A induced photosensitivity of yeast cells. (**a**) Overnight pre-cultures were inoculated in liquid YPD media (2 × 10^5^ cells/mL), and grown for 2 h before the EGCG solutions were added (final concentration, 10 μg/mL). Cell suspensions were exposed for 6 rounds of 10 s pulses of UV-A (365 nm) followed by 10 s breaks. Following the UV-A exposure, cells were incubated in the dark, and cell growth was assessed after 24 h relatively to wild type (WT) cells grown in YPD in the dark. Each determination was repeated three times on different days, with no significant variations (*p* < 0.05). Values are expressed as the mean ± standard deviation (SD) of duplicate determinations of three independent experiments (n = 6). (**b**) Effect of EGCG on the UV-A sensitivity of *yap1Δ* cells grown on top of agar plates. *Mid-log phase cells* were suspended at cell density of 1 × 10^5^ cells/mL in 5 mL of molten soft agar YPD (0.7% agar, 50 °C) containing 10 μg/mL EGCG (added from 10 mg/mL solutions) and spread on YPD-agar plates (9 cm diameter) containing the same concentration of the chemical. EGCG solutions were placed (0.1 mM, 15 μL) onto the surface of the top agar, then plates were subjected to six rounds of UV pulses (10 s each, followed by 10 s breaks). The halo of the growth zone around the spotted sample was observed after three days incubation at 28 °C in the dark. The experiments were repeated on three different days and the results were similar. One representative set of plates is shown. For controls, the same amount of the carrier solvents as in the EGCG experiments were used.

Cells were incubated in the dark for 2 h before being exposed to 6 rounds of 10 s pulses of UV (365 nm) followed by 10 s break. Following the UV-exposure, cells were returned to the incubator, and cell growth was determined (OD_660_) after 24 h incubation in the dark (Figure 6a). 

As the UV-A rays could be reflected/absorbed by the liquid media in which the cells were grown, we also irradiated cells spread onto the surface of agar plates. This time, the six rounds of UV-A pulses completely killed the cells, albeit the agar medium had been supplemented with EGCG (10 μg/mL). Placing 15 μL of EGCG solutions (100 μM, from 10 mM stock prepared in 99% EtOH or DMSO) completely rescued the *yap1Δ* sensitivity to UV light, while the carrier solvents alone or EGCG in water had no obvious effect (Figure 6b). These observations suggest that EGCG in EtOH or DMSO can exert a strong protective action against UV-A irradiation.

## 3. Experimental

### 3.1. Preparation of GTE

Coarse powder of dried green tea leaves were obtained from *S. C. Laboratoarele Fares Bio Vital S. R. L* (Orastie, Romania). The powder (2 g/sample) was additionally ground in a mortar under liquid nitrogen, then extracted twice with 5 mL solvent (water, aqueous ethanol, absolute ethanol or DMSO) in brown glass flasks under nitrogen. The flasks were stirred for 6 h in the dark at room temperatures. The extracts were centrifuged and the supernatant was sterilized by filtration (pore size 0.22 μm; Merck Millipore, Darmstadt, Germany). The extracts were adjusted to 2 mg/mL total polyphenolic content (TPC) with the corresponding solvent, aliquoted in hermetic and opaque flasks, and kept at −22 °C until further used. Unless otherwise stated, all chemicals were purchased from Sigma.

### 3.2. Determination of TPC

Total polyphenol content (TPC) of the extracts was determined with Folin-Ciocalteu reagent [25]. An aliquot (100 μL) of the sample prepared above was mixed with Folin-Ciocalteu reagent (50 μL) in ultra-pure water (0.5 mL) and vortexed. An aqueous solution of Na_2_CO_3_ (200 g/L, 0.6 mL) was added and the mixture was stored for 30 min at room temperature in darkness before the absorbance was measured at 760 nm on an JASCO V-630 UV-Vis spectrophotometer (Tokyo, Japan) using gallic acid as control and H_2_O as blank. Total polyphenols in the extracts were adjusted to 2 mg gallic acid equivalents/mL using the corresponding solvent.

### 3.3. Strains and Culture Conditions

The *S. cerevisiae* strains used in this study were isogenic to the “wild-type” (WT) parental strain BY4741 (*MAT***a**; *his3Δ1*; *leu2Δ0*; *met15Δ0*; *ura3Δ0*) [26]. The knock-out mutant strains used are listed in Table 1. All strains were obtained from EUROSCARF (European *S. cerevisiae* Archive for Functional Analysis, Institute of Molecular Biosciences Johann Wolfgang Goethe-University Frankfurt, Germany). Cell storage, growth and manipulation were done as described [27]. Strains were grown in standard YPD (yeast extract-polypeptone-dextrose). For solid media, 2% agar was used. 

### 3.4. Growth Assessment

All growth experiments carried out under illumination (32 W/m^2^) were paralleled by duplicate experiments carried out in the dark. 

#### 3.4.1. Growth in Liquid Media

Overnight pre-cultures were inoculated in fresh media at density 2 × 10^5^ cells/mL, then cells were incubated with shaking (200 rpm) at 28 °C for two hours before being used for various tests. The cell growth in liquid media was monitored at time intervals by determining the optical density of cellular suspension at 660 nm (Shimadzu UV-VIS spectrophotometer, UV mini 1240, Kyoto, Japan) as described [28]. 

#### 3.4.2. Cell Growth Spot Assay

Fresh cell cultures of OD_660_ ≈ 1 were diluted 10-, 100-, 1,000- and 10,000- fold and stamped on various agar plates using a replicator. Plates were photographed after 2–4 days incubation at 28 °C. 

#### 3.4.3. Halo Assay

The halo assay was performed on YPD-soft agar plates containing the tested chemicals [29]. The indicator cells were suspended at cell density of 1 × 10^5^ cells/mL in molten soft agar YPD (0.7% agar, 50 °C) containing the tested chemical, and spread on YPD-agar plate containing the same concentration of the chemical. After the solidification, GTEs were placed (15 μL, from 0.1 mM sterile stocks) onto the surface of the top agar. The halo of the growth zone around the spotted sample was observed after three days incubation at 28 °C.

### 3.5. UVA Irradiation of Cells

Cell suspentions pre-treated with EGCG for two hours (5 × 10^5^ cell/mL) in 48 well plates (100 μL/well) were irradiated with an UV-A lamp (Vilber Lourmat, Marne-la-Valée, France) emitting ultraviolet rays at 365 nm, which delivered uniform irradiation at a distance of 20 cm (10 mW/cm^2^). After UVA exposure, the cells were fed with 100 μL fresh medium containing EGCG, and further incubated with shaking in the dark at 28 °C until further analysis. In the hallo assay, the cells were suspended at cell density of 1 × 10^5^ cells/mL in 5 mL of molten soft agar YPD containing 10 μg/mL EGCG (added from 1 mg/mL solutions) and spread on YPD-agar plates (9 cm diameter) containing the same concentration of the chemical. After solidification of the top agar, EGCG solutions were placed (0.1 mM, 15 μL) onto the surface of the top agar, then plates were subjected to six rounds of UV pulses (10 s each, followed by 10 s break). The halo of the growth zone around the spotted sample was observed after three days incubation at 28 °C in the dark. 

### 3.6. Reproducibility of the Results

All experiments were repeated independently on three different days. For each individual experiment values were expressed as the mean ± standard deviation (SD) of duplicate determinations on three independent days (n = 6). Multiple comparisons were performed with *S*tudent’s *t*-test. The differences were considered to be significant when *p* < 0.05. Data analysis was performed with Statistical Package for Social Science 15.0 (SPSS 15.0) for Windows. The observed trends were fully consistent among the independent experiments. For visual results (photographs), a representative example is shown. 

## 4. Conclusions

The GTEs as well as their main chemical component EGCG had opposite effects upon yeast cell growth under light exposure, depending on the solvent used. While water or aqueous EtOH solutions augmented the sensitivity of yeast cells towards visible light, the 99% EtOH or DMSO solutions conferred protection under the same irradiation conditions. Moreover, similar deleterious/protective effects were noticed under ^1^O_2_ generation conditions, such as UV-A irradiation, or illumination in the presence of photosensitizers. These data may have technological implications, by providing a easily-reproducible and cost effective model for testing GTEs targeted for various purposes, from antimicrobial agents (combining water-based GTEs with illumination) to sunscreen cosmetics (supplemented with GTEs obtained in less-polar-than-water solvents).
